# Characterization of *Phytochrome-Interacting Factor* Genes in Pepper and Functional Analysis of *CaPIF8* in Cold and Salt Stress

**DOI:** 10.3389/fpls.2021.746517

**Published:** 2021-10-25

**Authors:** Youxin Yang, Yelan Guang, Feng Wang, Yue Chen, Wenting Yang, Xufeng Xiao, Sha Luo, Yong Zhou

**Affiliations:** ^1^Jiangxi Key Laboratory for Postharvest Technology and Nondestructive Testing of Fruits and Vegetables, Collaborative Innovation Center of Post-Harvest Key Technology and Quality Safety of Fruits and Vegetables, College of Agronomy, Jiangxi Agricultural University, Nanchang, China; ^2^College of Horticulture, Shenyang Agricultural University, Shenyang, China; ^3^Key Laboratory of Crop Physiology, Ecology and Genetic Breeding, Ministry of Education, Jiangxi Agricultural University, Nanchang, China; ^4^College of Bioscience and Bioengineering, Jiangxi Agricultural University, Nanchang, China

**Keywords:** pepper, *phytochrome-interacting factor* (*PIF*), expression profile, salt stress, cold stress

## Abstract

As a subfamily of basic helix-loop-helix (bHLH) transcription factors, phytochrome-interacting factors (PIFs) participate in regulating light-dependent growth and development of plants. However, limited information is available about PIFs in pepper. In the present study, we identified six pepper *PIF* genes using bioinformatics-based methods. Phylogenetic analysis revealed that the PIFs from pepper and some other plants could be divided into three distinct groups. Motif analysis revealed the presence of many conserved motifs, which is consistent with the classification of PIF proteins. Gene structure analysis suggested that the *CaPIF* genes have five to seven introns, exhibiting a relatively more stable intron number than other plants such as rice, maize, and tomato. Expression analysis showed that *CaPIF8* was up-regulated by cold and salt treatments. *CaPIF8*-silenced pepper plants obtained by virus-induced gene silencing (VIGS) exhibited higher sensitivity to cold and salt stress, with an obvious increase in relative electrolyte leakage (REL) and variations in the expression of stress-related genes. Further stress tolerance assays revealed that *CaPIF8* plays different regulatory roles in cold and salt stress response by promoting the expression of the *CBF1* gene and ABA biosynthesis genes, respectively. Our results reveal the key roles of *CaPIF8* in cold and salt tolerance of pepper, and lay a solid foundation for clarifying the biological roles of *PIFs* in pepper and other plants.

## Introduction

Plants have developed multiple signaling transduction processes for regulating growth and development, and modulating complex molecular mechanisms by driving transcriptional or translational changes in transcription factors under diverse environmental conditions (Haak et al., 2017; Yang et al., 2019). Light provides photosynthate and energy through photosynthesis as well as acts as an essential environmental signal that participates in plant development and stress response. Plants perceive light via photoreceptors and rapidly respond to light intensity, quality, and photoperiod (Galvão and Fankhauser, 2015; Li and Mathews, 2016; Roeber et al., 2021). Phytochromes (phys) are red (R)/far-red (FR) light photoreceptors synthesized in cytoplasm and can be translocated to the nucleus for mediating light-regulated modulation of plant growth and development by interacting with various downstream signal transduction elements (Klose et al., 2015; Pham et al., 2018; Paik and Huq, 2019). There are five phytochromes from phytochrome A (phyA) to phyE in *Arabidopsis*, and their regulatory mechanisms exhibit distinct characteristics (Wang and Wang, 2015; Pham et al., 2018). Specially, phytochrome-interacting factors (PIFs) are a subfamily of basic helix-loop-helix (bHLH) transcription factors acting as the downstream targets of phytochromes. Under light exposure, phytochromes undergo physical interactions with PIFs and further inhibit their functions by promoting the phosphorylation and ubiquitin proteasome-dependent proteolysis and/or sequestration of PIFs (Shen et al., 2005; Al-Sady et al., 2006; Park et al., 2018).

PIF proteins possess two conserved domains: bHLH and APB (active phyB-binding), and some PIFs have an additional APA (active phyA-binding) domain (Pham et al., 2018). In *Arabidopsis*, the APA and APB domains are responsible for the physical interaction of PIFs with phyA and phyB, respectively. PIF proteins are widely distributed from bryophytes to angiosperms, with only one member in *Marchantia polymorpha* (Inoue et al., 2016), six in *Physcomitrella patens* (Possart et al., 2017), six in *Oryza sativa* (Nakamura et al., 2007), seven in *Zea mays* (Zhang et al., 2018; Wu et al., 2019), seven in *Malus domestica* (Zheng et al., 2020), eight in *Arabidopsis thaliana* (Lee and Choi, 2017), eight in *Solanum lycopersicum* (Rosado et al., 2016), 11 in *Populus trichocarpa* (Ding et al., 2021), and 12, 16, and 30 in *Brassica rapa*, *B. oleracea*, and *B. napus*, respectively (Li W. et al., 2021).

PIFs have been shown to be a negative regulator in the light signal transduction pathway that can promote skotomorphogenic development and suppress photomorphogenesis under dark conditions. In *Arabidopsis*, most PIFs redundantly promote skotomorphogenesis partly by promoting cell elongation, and mutation of *PIF1*, *PIF3*, *PIF4*, and *PIF5* (usually named as *pifq*) would result in a striking constitutive photomorphogenesis phenotype in the dark (Leivar et al., 2008; Shin et al., 2009; Leivar and Monte, 2014). Unlike that of these PIFs, mutation of *PIF2* would lead to elongated hypocotyls and small cotyledons under continuous R and FR light, indicating that *PIF2* acts as a positive regulator in photomorphogenesis (Luo et al., 2014). In addition, AtPIF1, AtPIF3, AtPIF4, AtPIF5, and AtPIF7 promote the elongation of hypocotyl in response to red light, while AtPIF8 promotes the elongation of hypocotyl in response to blue and/or far-red light but not to red light (Pham et al., 2018; Oh et al., 2020). AtPIF4 and AtPIF5 not only act downstream of cryptochromes to mediate hypocotyl elongation in response to low blue light (Pedmale et al., 2016), but also participate in the UV-B photoreceptor UVR8 mediated hypocotyl elongation regulation (Tavridou et al., 2020).

PIFs can bind to a core G-box DNA-sequence motif (CACGTG) or PIF binding E-box (CANNTG) in the promoters of a great variety of target genes to activate or repress their expression (Zhang et al., 2013; Leivar and Monte, 2014; Luo et al., 2014; Sakuraba et al., 2017; Xi et al., 2021). Besides the vital role in photomorphogenesis, PIFs are also involved in the regulation of many other cellular processes, including fruit ripening, flowering time, and hormone response (Paik et al., 2017). For example, *Arabidopsis* PIFs have important functions in shade avoidance response (SAS) mainly through auxin biosynthesis and auxin signaling (Hornitschek et al., 2012; Hersch et al., 2014; Pacín et al., 2016; Jia et al., 2020), as well as via other phytohormones including gibberellin (GA) and brassinosteroid (BR) (Feng et al., 2008; Sorin et al., 2009; Xie et al., 2017; Hayes et al., 2019; Wang X. et al., 2020). Additionally, PIF1, PIF3, PIF4, PIF5, and PIF7 are necessary for the phy-mediated seasonal regulation of hypocotyl elongation in *Arabidopsis* (Leivar et al., 2020). In *Populus*, both PtPIF4 and PtPIF8 have conserved and redundant functions in SAS, but only PtPIF8 participates in regulating seasonal growth (Ding et al., 2021). *Rosa chinensis* RcPIFs function as a suppressor of flowering in response to light intensity by interacting with RcCO and thus impair the binding of RcCO to the *RcFT* promoter (Sun et al., 2021). AtPIF4 and AtPIF5 can activate dark-, age-, and heat-induced leaf senescence via regulation of *ORESARA 1* (*ORE1*) and other known senescence-associated genes (SAGs) (Sakuraba et al., 2014; Song et al., 2014; Kim et al., 2020; Li N. et al., 2021). PIFs are also involved in the regulation of biotic and abiotic stress responses in plants. *Arabidopsis* PIFs were found to have redundant control over plant defense response to *Botrytis cinerea* (Xiang et al., 2020). Both PIF4 and PIF7 have central functions in thermomorphogenesis downstream of phyB, and PIF4 plays a more crucial role than PIF7 in promoting auxin response during thermomorphogenesis in *Arabidopsis* (Koini et al., 2009; Zhu et al., 2016; Chung et al., 2020; Fiorucci et al., 2020). Maize *ZmPIF3.1* can confer drought resistance by regulating stomatal closure under the mediation of abscisic acid (ABA) in rice (Gao et al., 2018). In addition, overexpression of apple *MdPIF3* would decrease the cold tolerance but promote drought resistance of both *Arabidopsis* plants and apple callus (Zheng et al., 2021).

Pepper (*Capsicum annuum* L.) is an economically important vegetable crop worldwide for its high contents of many essential nutrients. However, its growth and development are usually negatively influenced by various stresses, causing a serious loss of its yield and quality (Guo et al., 2015; Zhang R.X. et al., 2020). Identification of resistance genes in pepper will provide valuable guidance and resources for breeding to enhance its yield and quality under stress conditions. Although previous studies have revealed the functions of PIFs in regulating the development and stress response of plants, the functions of PIFs in pepper remain poorly understood. In this study, six *PIF* family members were identified from the pepper genome, and their phylogenetic relationship, gene structure, conserved motif, and expression profile were analyzed. Besides, virus-induced gene silencing (VIGS) of *CaPIF8* was found to reduce the tolerance of pepper to cold and salt stress. Our results provide important information for further revealing the role of *CaPIF*s in pepper.

## Materials and Methods

### Identification of Proteins Encoded by the *Phytochrome-Interacting Factor* Genes in Pepper

To screen the PIF proteins in pepper, all capsicum proteins were acquired from three public databases: PepperHub server,^1^ Solanaceae Genome Database,^2^ and NCBI.^3^ Then, the amino acid sequences of AtPIFs were obtained from TAIR,^4^ and used as queries to search for the homologous sequences from the capsicum proteome. The candidate pepper PIFs were submitted to SMART^5^ and InterPro^6^ for checking the presence of conserved bHLH and APB domains.

### Protein Properties and Sequence Analysis of Pepper *Phytochrome-Interacting Factors*

The molecular weight (MW) and isoelectric point (pI) of the candidate PIFs were calculated with ExPasy.^7^ In order to study the evolutionary relationships of the PIFs between pepper and other plant species, the protein sequences of PIFs from pepper, *Arabidopsis*, tomato, rice, and maize were aligned by Clustal Omega, and the alignment results were used to create an NJ (neighbor-joining) phylogenetic tree with the MEGA7.0 software using the pairwise deletion option, Poisson correction and 1,000 bootstrap replicates. The online MEME tool was used to search the conserved motifs of the pepper, *Arabidopsis*, tomato, rice, and maize, and the results were visualized by TBtools (Chen et al., 2020). The online tool Gene Structure Display Server v2.0^8^ was employed to analyze the gene structures of *PIFs* from different plant species.

### Subcellular Localization of *CaPIF8*

The full-length coding region without the stop codon (1,416 bp) of *CaPIF8* (XM_016689836.1) was cloned into the Super1300-GFP vector between the X*ba* I and K*pn* I sites. The pSuper1300:CaPIF8:GFP vector was transformed into *Agrobacterium tumefaciens* strain GV3101. The pSuper1300:CaPIF8:GFP and pSuper1300:GFP (as control) were grown overnight, re-suspended in the induction medium (10 mM MgCl_2_, 10 mM MES, and 200 μmol L^–1^ acetosyringone), and injected into *Nicotiana benthamiana*. GFP fluorescence was photographed using a confocal laser-scanning microscope (Nikon C2-ER, Tokyo, Japan).

### Expression Profiling of *CaPIF* Genes by RNA-Seq Data

To obtain the expression profiles of *CaPIF* genes in various tissues including bud, flower, leaf, root and stem, transcriptomic data of pepper cultivar “Zunla” were obtained from publicly available GEO database under the accession numbers of GSE45037 and GSE45154 (Qin et al., 2014). The genome-wide transcriptome data of an elite breeding pepper (*Capsicum annuum*) line 6,421 treated with 200 mM NaCl and 10°C were obtained under PepperHub server (see text footnote 1) (Liu et al., 2017). The gene expression levels were measured following the TopHat/Cufflinks pipeline based on the FPKM (fragments per kilobase of exon per million fragments mapped) values according to the previous reports (Haq et al., 2019; Wu et al., 2020). The heatmaps of the gene log2 transformed values were generated with TBtools software.

### Plant Materials and Growth Conditions

Hangjiao12 (*Capsicum annuum* L.) seedlings were planted in a greenhouse under 25/18°C (day/night) temperature with a 12 h photoperiod. For cold stress, TRV2:CaPIF8 and TRV2:00 seedlings at the four-leaf stage were placed in 4°C incubator. RNA samples were collected from leaves after 6 h of cold treatment. For salt stress, 300 mM NaCl (90 mL per plant) was used to irrigate the seedlings. RNA samples were collected from the leaves after 12 h of salt treatment. Harvested samples were rapidly frozen by using liquid nitrogen and stored at –80°C.

### Virus-Induced Gene Silencing Experiment in Pepper

Tobacco rattle virus (TRV) based VIGS was used to generate *CaPIF8*-silenced pepper plants. Partial cDNA fragment of *CaPIF8* (300 bp) was amplified with the VIGS tool.^9^ The PCR-amplified fragment was cloned into the pTRV2 vector by using the restriction sites of E*co*R I and X*ho* I. The empty vector of TRV2:00 served as the control, and the TRV2:CaPDS (phytoene desaturase gene) vector was set as positive control due to the photobleaching phenotype, and above TRV2 and TRV1 were transformed into *Agrobacterium tumefaciens* strain GV3101. Two-week-old pepper seedlings were used for silencing the *PIF8* gene according to the previously described method (Yang et al., 2015).

### Total RNA Extraction and Quantitative Real-Time PCR Analysis

Total RNA extraction was conducted with the total RNA Miniprep Kit (Axygen Biosciences, Union City, CA, United States). Approximately 5 μg of RNA was reverse transcribed with the ReverTra Ace qPCR-RT Kit (Toyobo, Japan) according to the manufacturer’s protocol. qRT-PCR was carried out in triplicate using the iCycler iQ^TM^ Real-time PCR Detection System (Bio-Rad, Hercules, CA, United States) with the procedures as previously described (Yang et al., 2019). The pepper *actin* gene was used as an internal control. The primers of *actin* and *CaPIF* genes are listed in Supplementary Table 1.

### Determination of Electrolyte Leakage and Abscisic Acid Content

To assess membrane permeability, electrolyte leakage assays were performed as described previously by using a conductivity meter (ST3100C, Changzhou, China). Relative electrolyte leakage (REL) was calculated based on the previously described method (Haq et al., 2019; Zhang H. et al., 2020). To determine the ABA content, 0.25 g of frozen leaves was ground into powder in liquid nitrogen. ABA was extracted in 1.5 mL of 80% aqueous methanol overnight at 4°C. After centrifugation, the supernatant was removed and extracted twice with 2 mL petroleum ether, the pH was adjusted to 2–3 with citric acid, and 2 mL ethyl acetate was used to extract the supernatant twice. The upper organic phase was transferred to a new EP tube, blown dry under the flow of N_2_ (gas). The dried samples were dissolved and mixed with 0.2 mL of mobile phase, and filtered through a needle filter. ABA content was analyzed using a high-performance liquid chromatography (Waters 2695, Milford, MA, United States).

### Statistical Analysis

Significant differences (*P* < 0.05) were tested by Duncan’s test in the statistical product and service solution (SPSS) software between the data from the control and treatments, and different letters were used to indicate differences.

## Results

### Genome-Wide Identification and Sequence Analysis of Pepper *Phytochrome-Interacting Factors*

In total, six *PIF* genes were identified from the pepper genome, which were designated as *CaPIF1*, *CaPIF3*, *CaPIF4*, *CaPIF7a*, *CaPIF7b*, and *CaPIF8*. Table 1 presents the physiological and biochemical properties of these six *CaPIF* genes. The gene lengths of *CaPIFs* varied from 1,950 to 11,887 bp, and the amino acid sequence lengths were from 342 to 633 aa. The predicted pI of various proteins ranged from 5.55 to 7.31 (Table 1).

**TABLE 1:** Characteristics of *phytochrome-interacting factor* (*PIF*) genes in pepper.

Name	ID	Chromosome	gDNA (bp)	Length (aa)	MW (kDa)	pI	Functional domains
*CaPIF1*	Capana09g001161	9	11,887	547	59.498	5.55	bHLH, APB, APA
*CaPIF3*	Capana08g001686	8	3,328	633	68.175	7.23	bHLH, APB, APA
*CaPIF4*	Capana07g001126	7	2,321	485	53.847	6.70	bHLH, APB
*CaPIF7a*	Capana03g000857	3	2,449	460	50.680	7.31	bHLH, APB
*CaPIF7b*	Capana00g004742	0	1,950	342	38.608	6.68	bHLH, APB
*CaPIF8*	Capana01g003548	1	4,541	468	50.692	6.27	bHLH, APB

*bHLH, basic helix-loop-helix; APB, active phyB-binding domain, APA, active phyA-binding domain.*

**FIGURE 1:**
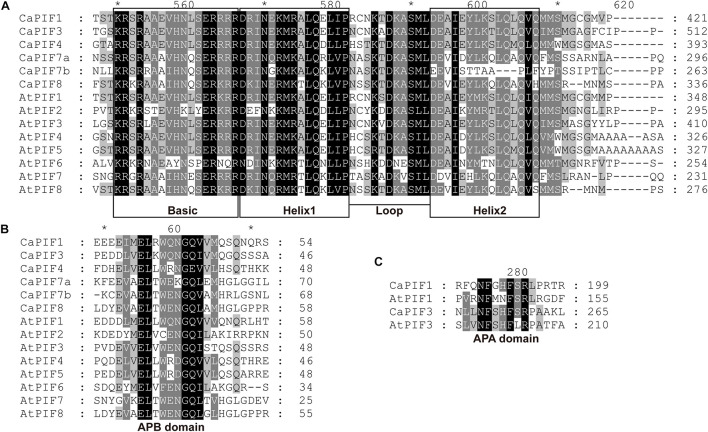
Multiple sequence alignment of the conserved bHLH **(A)**, APB **(B)**, and APA **(C)** domains of PIFs from pepper and *Arabidopsis*. Multiple sequence alignment was performed with Clustal Omega and displayed with the GeneDoc software. Asterisks were automatically generated by the GeneDoc software.

To analyze the residue conservation features in these CaPIFs, the full-length CaPIFs and AtPIFs were aligned by Clustal Omega. As a result, the bHLH and APB domains were found in all six CaPIF proteins, with the exception of CaPIF7b, which contained an incomplete Helix2 in the bHLH domain (Table 1 and Figure 1). In addition, CaPIF1 and CaPIF3 contained an additional APA domain, which was also observed in PIF proteins from other plants, such as tomato SlPIF1a, SlPIF1b, and SlPIF3 (Rosado et al., 2016), *Arabidopsis* AtPIF1 and AtPIF3 (Lee and Choi, 2017), and apple MdPIF1–MdPIF5 (Zheng et al., 2020).

### Phylogenetic Analysis and Conserved Motifs of *Phytochrome-Interacting Factors* in Pepper and Other Plant Species

**FIGURE 2:**
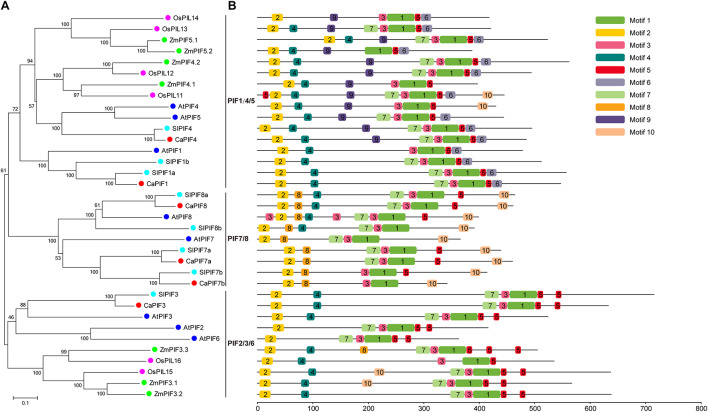
Phylogenetic analysis and conserved motif analysis of the PIF proteins of pepper, *Arabidopsis*, tomato, rice, and maize. **(A)** Evolutionary relationships of PIF proteins in pepper, *Arabidopsis*, tomato, rice, and maize. The phylogenetic tree was created with the neighbor-joining method and 1,000 bootstrap replications conducted in MEGA 7.0. The accession numbers of PIFs used to construct the phylogenetic tree are listed in Supplementary Table 2. **(B)** Distribution of the 10 conserved motifs in the PIF proteins of pepper, *Arabidopsis*, tomato, rice, and maize indicated by the analysis with MEME. Motifs are colored by different boxes and their sequences are listed in Supplementary Table 3.

To elucidate the evolution of PIFs in pepper and other plant species, a NJ phylogenetic tree was created on the basis of multiple sequence alignment of full-length PIF protein sequences from pepper, *Arabidopsis* (Lee and Choi, 2017), tomato (Rosado et al., 2016), rice (Nakamura et al., 2007), and maize (Wu et al., 2019). In the phylogenetic tree, these PIF proteins could be divided into three distinct groups, namely PIF1/4/5, PIF2/3/6, and PIF7/8, and CaPIFs displayed a closer relationship with tomato SlPIFs and *Arabidopsis* AtPIFs than with rice OsPIFs and maize ZmPIFs (Figure 2A).

Conserved motif analysis was also conducted with the MEME tool, resulting in the identification of 10 conserved motifs (Figure 2B). Among these motifs, motif 1, and motif 2 were annotated as the bHLH and APB domains, which are present in all PIF proteins. In addition, motifs 3, 5, and 7 were also widely distributed in these PIF proteins. However, several motifs were unique to specific groups. For example, motif 9 was identified specifically in PIF4/5 members. Motif 8 was only observed in ZmPIF3.3 and all PIF7/8 members, and ZmPIF3.3 was lack of motif 10, which was widely found in PIF7/8 group (Figure 2B). In addition, PIF7 proteins were lack of motif 4, which was widely present in PIF proteins of other groups, except for OsPIL14, AtPIF2, and AtPIF6 (Figure 2B).

### Gene Structure of *Phytochrome-Interacting Factor* Genes in Pepper and Other Plant Species

**FIGURE 3:**
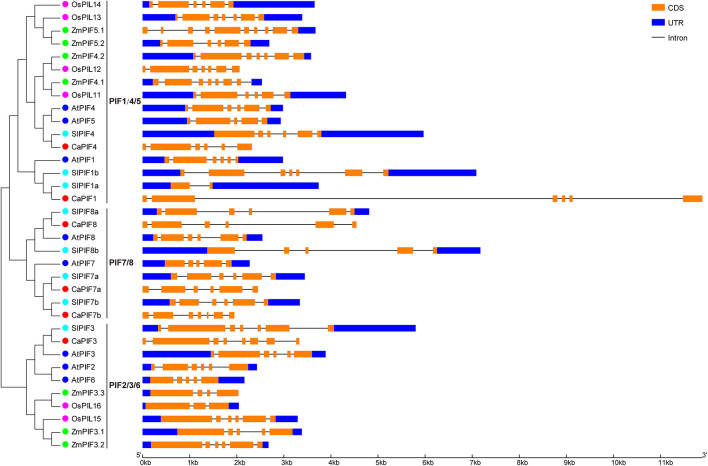
Structural analysis of *PIF* genes from pepper and other plant species according to the phylogenetic relationship. The CDS and untranslated region (UTR) are presented by orange and blue boxes, respectively.

We also analyzed the structure of the *PIF* genes from pepper and other plant species. As a result, most of the *PIF* genes possessed 4–6 introns, especially those from dicots. The number of introns in *CaPIF* genes ranged from five to seven, while that of *PIF* genes in other plants varied more greatly, ranging from one (*SlPIF1a*) to nine (*ZmPIF5.1*) (Figure 3). In addition, some closely related *PIF* genes exhibited similar gene structures in intron number and CDS length, such as *ZmPIF4.2* and *OsPIL12*, *SlPIF8a* and *CaPIF8*, *SlPIF7a* and *CaPIF7a* (Figure 3).

### Expression Profiles of *CaPIF* Genes in Different Tissues

**FIGURE 4:**
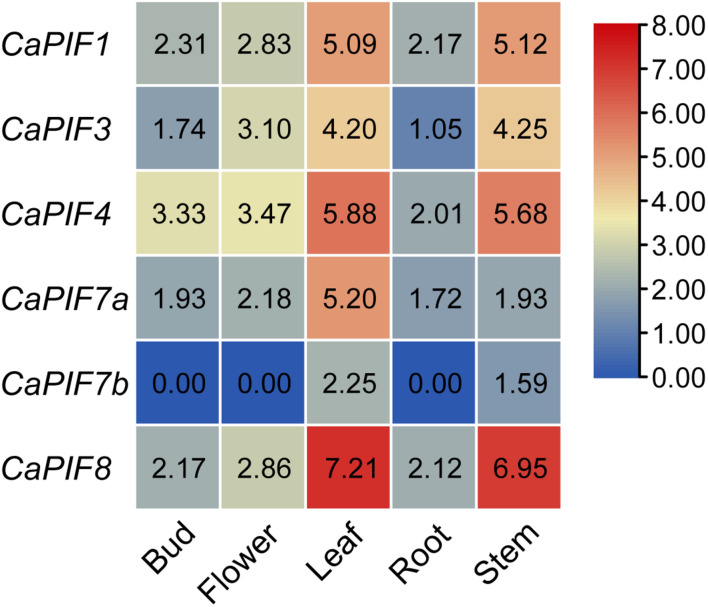
Relative expression levels of *CaPIF* genes in different tissues based on RNA-seq data. RNA-seq data of five tissues in pepper cultivar “Zunla” were used to analyze the expression pattern. Roots, stems, and leaves were collected from plants at the full-bloom stage, and the unopened flower buds (buds) and fully open flowers (flowers) were collected from mature plants. The heat map was drawn in log2 transformed expression values.

To elucidate the possible functions of *CaPIF* genes, their tissue expression profiles were analyzed with publicly available transcriptomic data. As a result, *CaPIF1*, *CaPIF3*, *CaPIF4*, *CaPIF7a*, and *CaPIF8* were constitutively expressed in all the tissues tested, while *CaPIF7b* was only expressed in leaves and stems (Figure 4). Notably, all *CaPIF* genes had high expression in the leaves and stems, but there were some differences among *CaPIF* genes. For example, the expression of *CaPIF8* was much higher than that of other *PIF* genes in leaves and stems. Only one gene, *CaPIF7a*, showed a higher expression level in the leaves than in the stems, and other genes exhibited no obvious difference in expression between the two tissues (Figure 4).

### Expression Patterns of *CaPIFs* in Response to Cold and Salt Stress

**FIGURE 5:**
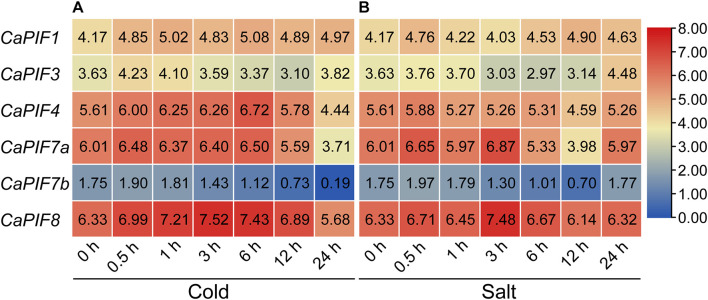
Expression profiles of *CaPIF* genes under cold **(A)** and salt **(B)** stress based on RNA-seq data. Cold and salt stress treatments were applied on 40-day old seedlings of an elite breeding pepper line 6,421 treated with 10°C and 200 mM NaCl, respectively, and leaf samples were collected at 0, 0.5, 1, 3, 6, 12, and 24 h post treatment. Numbers in the boxes show the expression based on log2 (FPKM +1) values.

To investigate the possible functions of *CaPIFs* under abiotic stresses, we examined their expression profiles under cold and salt stress based on public RNA-seq data. Under cold stress treatment, *CaPIF4* and *CaPIF8* displayed up-regulated expression at certain time points (3 h and/or 6 h), but the expression decreased at 24 h (Figure 5A). The expression of *CaPIF7a* and *CaPIF7b* was down-regulated at the later time points (24 h) (Figure 5A). Under salt stress treatment, *CaPIF4*, *CaPIF7a*, and *CaPIF7b* were down-regulated at 12 h, while *CaPIF8* was up-regulated at 3 h (Figure 5B). Since *CaPIF8* showed noticeably high expression under cold and salt stress, and its homolog in citrus (*CsPIF8*) was found to enhance the tolerance of citrus to cold stress (He et al., 2020), we then chose *CaPIF8* for further research in cold and salt stress.

### Subcellular Localization of *CaPIF8*

**FIGURE 6:**
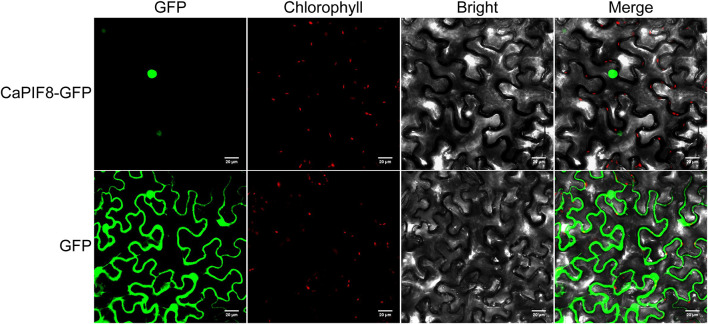
Subcellular localization of CaPIF8 in *Nicotiana benthamiana* epidermal cells. The subcellular localization of CaPIF8-GFP in *N. benthamiana* epidermal cells was visualized using confocal laser scanning microscopy.

For subcellular localization analysis of CaPIF8, the pSuper1300:CaPIF8:GFP and pSuper1300:GFP (as control) vectors were infiltrated into *Nicotiana benthamiana* epidermal cells by transient *Agrobacterium*-mediated transformation. Confocal laser scanning microscopy revealed that the GFP fluorescence of CaPIF8-GFP fusion protein was exclusively located in the nucleus, while that of 35S::GFP control was ubiquitously distributed in the whole cells (Figure 6), indicating that CaPIF8 is localized to the nucleus.

### Effect of *CaPIF8* Silencing on Cold Stress Tolerance of Pepper

**FIGURE 7:**
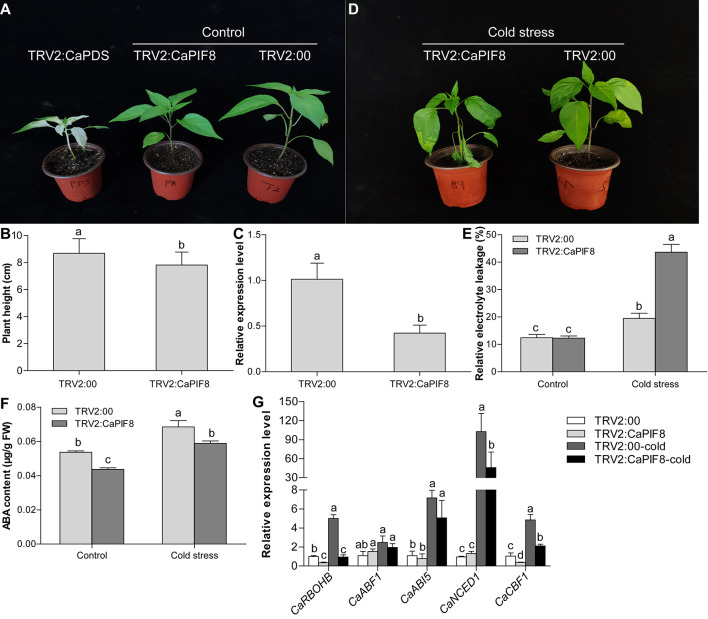
Effect of *CaPIF8* silencing on the cold stress tolerance of pepper. **(A)** Phenotypes of pepper plants after approximately 1 month of virus injection. TRV2:CaPDS, plants with the TRV2:CaPDS vector; TRV2:CaPIF8, *CaPIF8*-silenced plants; TRV2:00, control plants with the empty TRV2 vector. **(B)** Height of TRV2:00 and TRV2:CaPIF8 pepper plants before cold stress. **(C)** Relative expression of *CaPIF8* in leaves from *CaPIF8*-scilenced and control pepper plants. Leaf samples from TRV2:00 and TRV2:CaPIF8 plants were collected before cold stress. **(D)** Phenotypes of TRV2:00 and TRV2:CaPIF8 plants after 4°C cold stress for 3 days. **(E)** REL of TRV2:00 and TRV2:CaPIF8 plants. Leaf samples from TRV2:00 and TRV2:CaPIF8 plants were collected without and with cold stress for 3 days. **(F)** ABA content of TRV2:00 and TRV2:CaPIF8 plants. Leaf samples from TRV2:00 and TRV2:CaPIF8 plants were collected without and with cold stress for 12 h. **(G)** Relative expression levels of stress-related genes examined by qRT-PCR in leaves from TRV2:00 and TRV2:CaPIF8 plants without and with cold stress for 6 h. Each value is shown as mean ± standard error of three biological replicates, and the multiple comparison test for each gene was presented. Different letters stand for significant differences between TRV2:00 and TRV2:CaPIF8 plants without and with cold stress (Duncan’s test, *P* < 0.05).

Since the expression of *CaPIF8* markedly increased at specific time points under cold and salt treatments, it can be speculated that *CaPIF8* plays certain roles in stress response. Hence, we silenced *CaPIF8* in pepper plants by VIGS to study its possible role in cold and salt stress tolerance. After approximately 1 month of virus injection, the leaves of TRV2:CaPDS plants were photo-bleached, while those of plants infected with TRV2:00 and TRV2:CaPIF8 showed no obvious symptoms (Figure 7A). However, the TRV2:CaPIF8 plants had significantly lower height than the TRV2:00 plants (Figure 7B). The qRT-PCR results showed that the *CaPIF8* transcription in TRV2:CaPIF8 plants was reduced by 39% compared with that in TRV2:00 plants (Figure 7C), suggesting the successful silencing of *CaPIF8*.

To investigate the response of TRV2:CaPIF8 plants to cold stress, the TRV2:00 and TRV2:CaPIF8 plants were subjected to 4°C cold treatment for 3 days. After the treatment, the TRV2:CaPIF8 plants displayed severe growth retardation compared with the TRV2:00 plants, including serious wilting of leaves (Figure 7D). We also measured the REL and ABA level in leaves of the TRV2:00 and TRV2:CaPIF8 plants. Before cold stress treatment, no difference in REL was found between TRV2:00 and TRV2:CaPIF8 plants, while the TRV2:CaPIF8 plants exhibited markedly higher REL than the TRV2:00 plants after cold stress (Figure 7E). The ABA content in the TRV2:CaPIF8 plants was significantly lower than that in the TRV2:00 plants before and after cold stress treatment, though cold stress treatment led to increases in the ABA content of both plants (Figure 7F).

To elucidate the possible function of *CaPIF8* in cold stress response, the expression profiles of five stress-related genes were determined by qRT-PCR. As a result, cold treatment did not significantly change the expression of ABA responsive genes (*CaABF1* and *CaABI5*) in both TRV2:00 and TRV2:CaPIF8 plants (Figure 7G). However, the TRV2:CaPIF8 plants showed observably lower expression levels of *CaRBOHB*, *CaCBF1*, and ABA biosynthesis gene (*CaNCED1*) than the TRV2:00 plants with or without cold treatment (Figure 7G).

### Effect of *CaPIF8* Silencing on Salt Stress Tolerance of Pepper

**FIGURE 8:**
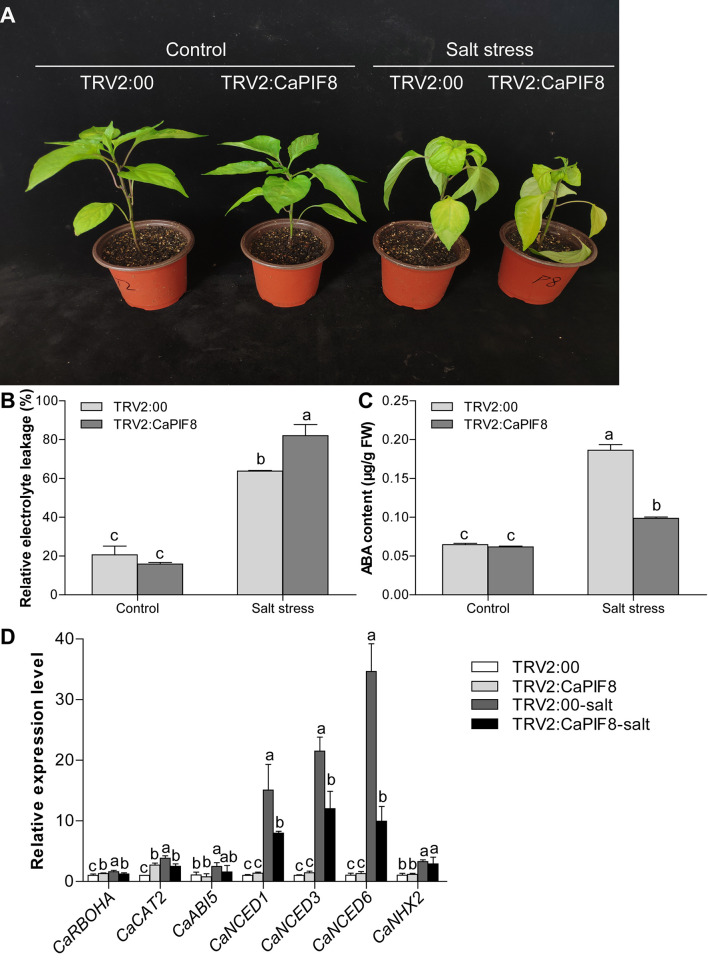
Effect of *CaPIF8* silencing on the salt stress tolerance of pepper. **(A)** Phenotypes of TRV2:00 and TRV2:CaPIF8 pepper plants without and with salt stress treatment. The TRV2:00 and TRV2:CaPIF8 plants were subjected to salt stress by irrigation of 300 mM NaCl solution for 7 days. **(B)** REL of TRV2:00 and TRV2:CaPIF8 plants. Leaf samples from TRV2:00 and TRV2:CaPIF8 plants were collected without and with salt stress for 7 days. **(C)** ABA content of TRV2:00 and TRV2:CaPIF8 plants. Leaf samples from TRV2:00 and TRV2:CaPIF8 plants were collected without and with salt stress for 3 days. **(D)** Relative expression levels of stress-related genes examined by qRT-PCR in leaves from TRV2:00 and TRV2:CaPIF8 plants without and with salt stress for 12 h. Each value is shown as mean ± standard error of three biological replicates, and the multiple comparison test for each gene was presented. Different letters stand for significant differences between TRV2:00 and TRV2:CaPIF8 plants without and with salt stress (Duncan’s test, *P* < 0.05).

Additionally, we tested the response of TRV2:CaPIF8 plants to salt stress. After salt stress treatment for 7 days, the TRV2:CaPIF8 plants exhibited more serious symptoms than TRV2:00 plants, including leaf shrinkage, yellowing, and even wilting (Figure 8A). Also, no significant difference was observed in REL and ABA content between TRV2:00 and TRV2:CaPIF8 plants without salt stress. Upon salt stress treatment, the TRV2:CaPIF8 plants exhibited remarkably higher REL (Figure 8B) and a nearly 50% decrease in ABA content (Figure 8C) relative to the TRV2:00 plants. As for the stress-related genes, *CaABI5* and *CaNHX2* showed no significant changes in expression with and without salt treatment in both TRV2:00 and TRV2:CaPIF8 plants (Figure 8D). Salt treatment increased the expression of *CaRBOHA*, *CaCAT2*, and ABA biosynthesis genes (*CaNCED1*, *CaNCED3*, and *CaNCED6*) in both TRV2:CaPIF8 and TRV2:00 plants, and the expression was much lower in TRV2:CaPIF8 plants than in TRV2:00 plants (Figure 8D).

## Discussion

### *Phytochrome-Interacting Factor* Genes in Pepper and Their Evolution

In this work, six *PIF* genes were identified from the pepper genome, and the number was comparable to that in many other plants, such as *P. patens* (six genes) (Possart et al., 2017), *O. sativa* (six genes) (Nakamura et al., 2007), *Z. mays* (seven genes) (Zhang et al., 2018; Wu et al., 2019), *M. domestica* (seven genes) (Zheng et al., 2020), *A. thaliana* (eight genes) (Lee and Choi, 2017), and *S. lycopersicum* (eight genes) (Rosado et al., 2016). Amino acid sequence alignment revealed that all the six CaPIF proteins harbor a conserved bHLH and an APB domain (Figures 1A,B), which is a typical feature of PIF proteins. However, CaPIF7b has an incomplete Helix2 in its bHLH domain, which may affect its function to interact with other proteins. Notably, an amino acid substitution (Q to E) in the APB domain was observed in CaPIF4 (Figure 1B), which was also identified in MdPIF8 (Zheng et al., 2020), suggesting that they possibly have similar functions. Similar to tomato SlPIF1s and SlPIF3 (Rosado et al., 2016), maize ZmPIF3s (Wu et al., 2019), Arabidopsis AtPIF1 and AtPIF3 (Leivar and Quail, 2011), CaPIF1 and CaPIF3 also contain the conserved functional APA domain (Figure 1C), which was found to play a vital role in modulating the interaction with phyA.

In the phylogenetic tree, PIF proteins from different plants (pepper, *Arabidopsis*, tomato, rice, and maize) could be divided as three distinct groups, and members of the same group have similar conserved proteins motif arrangements (Figure 2). PIF proteins did not cluster in a species-specific manner; however, CaPIFs had a much closer phylogenetic relationship with PIFs from dicots than with those from monocots (Figure 2A). Additionally, the PIF1/4/5 and PIF2/3/6 groups comprise PIF proteins from both dicots and monocots, while the PIF7/8 group exclusively includes PIF proteins from dicots (Figure 2A), indicating the evolution of the *PIF* genes after the monocotyledon–dicotyledon differentiation, and the *PIF* genes in the PIF7/8 group might have been derived from those in the PIF1/4/5 and PIF2/3/6 groups. Compared with *Arabidopsis*, pepper is lack of PIF2, PIF5, and PIF6 homologs (Table 1 and Figure 2A), and the intron numbers of *CaPIF* genes are relatively stable compared with those of *PIFs* in other plants (Figure 3). It can be speculated that pepper *PIF* genes might have undergone relatively small variations and gene loss during evolution.

### *CaPIF8* Plays Positive but Differential Roles in Cold and Salt Tolerance of Pepper

Besides their crucial functions in photomorphogenesis, PIFs were also found to either positively or negatively regulate the response to different environmental stresses. For instance, both *AtPIF1* and *AtPIF4* were upregulated under heat and cold stress conditions; *AtPIF6* was upregulated by ABA and salt stress; while *AtPIF7* was suppressed by cold stress. AtPIF1, AtPIF4, and AtPIF5 were destabilized by cold stress depending on phytochromes, and they negatively regulated freezing tolerance in *Arabidopsis* (Jiang et al., 2020). *AtPIF4* functions as negative regulator of salt tolerance by directly regulating the expression of genes associated with salt stress response, including *ORE1*, *SAG29*, and *JUNGBRUNNEN1 (JUB1)/ANAC042* (Sakuraba et al., 2017). *ZmPIF3.1* was significantly induced by drought and salt stress, and rice overexpressing maize *ZmPIF3.1* showed enhanced tolerance to drought and salt stress (Gao et al., 2015). *OsPIL14* overexpression or loss-of-function of the DELLA protein SLENDER RICE1 (*SLR1*) could also enhance the salt tolerance of rice seedlings by integrating light and GA signals (Mo et al., 2020). In this study, *CaPIF4*, *CaPIF7a*, *CaPIF7b*, and *CaPIF8* were differentially regulated by cold or salt stress, indicating their critical and different roles in regulating the response to the two stresses (Figure 5). Among these four genes, *CaPIF8* was induced under both cold and salt stress, suggesting that this gene may positively regulate plant resistance to cold or salt stress. To verify this inference, we generated *CaPIF8-*silenced pepper plants by using the VIGS technique. As a result, the TRV2:CaPIF8 plants exhibited more serious symptoms under cold and salt stress with increased REL than the TRV2:00 plants (Figures 7, 8). Compared with the TRV2:00 plants, the TRV2:CaPIF8 plants exhibited observable decreases in the expression of *CaRBOHB* under cold treatment, and *CaRBOHA* and *CaCAT2* under salt treatment, respectively (Figures 7G, 8D), suggesting that reactive oxygen species (ROS) homeostasis was affected in TRV2:CaPIF8 plants under cold or salt stress. As the citrus homolog of CaPIF8 and AtPIF8, CsPIF8 can enhance the tolerance of citrus to cold stress through the regulation of SOD activity and the expression of *CsSOD* by binding to the E-box in its promoter (He et al., 2020). Therefore, CaPIF8 may participate in the regulation of the ROS homeostasis under cold and salt stress.

Various adverse stresses can result in ABA accumulation in plants, which can regulate stress-related genes involved in plant adaptation to environmental stimuli (Vishwakarma et al., 2017; Zhou et al., 2018). Some *PIF* genes were reported to participate in plant response to multiple stresses by activating or inhibiting downstream genes in the ABA-dependent signaling pathway. For example, overexpression of either *ZmPIF3.1* or *ZmPIF3.2* in rice conferred ABA-dependent drought tolerance (Gao et al., 2015, 2018). *Myrothamnus flabellifolia MfPIF1* also positively regulates the response to drought and salt stress by activating the expression of both ABA biosynthesis and ABA-responsive genes (Qiu et al., 2020). In this study, the TRV2:CaPIF8 plants showed lower tolerance to salt stress with a significant decrease in ABA content (Figure 8). qRT-PCR analysis revealed that compared with those in the TRV2:00 plants, the ABA biosynthesis genes (*CaNCED1*, *CaNCED3*, and *CaNCED6*) were down-regulated but the ABA responsive gene showed no obvious change in TRV2:CaPIF8 plants (Figure 8D). Hence, we speculate that *CaPIF8* has a crucial function in salt stress tolerance by activating ABA biosynthesis genes and then enhancing the ABA level under salt stress.

The TRV2:CaPIF8 plants showed higher sensitivity to cold stress, but the expression of ABA responsive genes (*CaABF1* and *CaABI5*) had no obvious change when compared with those in the control. However, *CaCBF1* was observably down-regulated in TRV2:CaPIF8 plants with and without cold stress (Figure 7G). The *C-repeat binding factor* (*CBF*) genes were found to enhance low temperature tolerance of plants. A previous study has suggested that AtPIF4 and AtPIF7 can reduce freezing tolerance through direct repression of the *CBF* genes under long day conditions (Lee and Thomashow, 2012). *Arabidopsis* CBF1 can interact with PIF3 but not with PIF1, PIF4, and PIF5, and stabilize PIF3 and phyB protein under cold stress (Jiang et al., 2020). In tomato, *SlPIF4* can positively regulate low R/FR light-induced cold tolerance by inducing the *CBF* genes (Wang F. et al., 2020). Therefore, the decrease in cold stress tolerance of the TRV2:CaPIF8 plants may be mainly attributed to the significant decrease in the expression of the *CBF* genes.

In this study, six PIF members were identified in pepper, which were then classified into three distinct groups together with PIFs from other plants. Subsequently, their conserved domains, gene structures and expression profiles were analyzed. Amongst the identified *CaPIF* genes, *CaPIF8* was upregulated under cold and salt stress, and silencing of *CaPIF8* reduced the tolerance of pepper to cold and salt stress, suggesting that *CaPIF8* can positively regulate tolerance to cold and salt stress. *CaPIF8* enhances cold stress tolerance probably by inducing the *CBF* genes, while mediates salt stress tolerance by activating the ABA biosynthesis genes. Therefore, it can be inferred that *CaPIF8* plays differential roles in mediating cold and salt tolerance of pepper. Overall, these findings provide important information for further revealing the mechanisms through which *CaPIF8* controls the cold and salt stress response of pepper.

## Data Availability Statement

The datasets presented in this study can be found in online repositories. The names of the repository/repositories and accession number(s) can be found in the article/Supplementary Material.

## Author Contributions

YY, FW, and YZ conceived and designed the experiments. YY, YG, FW, YC, WY, XX, SL, and YZ performed the experiments. YY, YG, and YZ analyzed the data. YY and YZ wrote the manuscript. All authors have read and approved the manuscript.

## Conflict of Interest

The authors declare that the research was conducted in the absence of any commercial or financial relationships that could be construed as a potential conflict of interest.

## Publisher’s Note

All claims expressed in this article are solely those of the authors and do not necessarily represent those of their affiliated organizations, or those of the publisher, the editors and the reviewers. Any product that may be evaluated in this article, or claim that may be made by its manufacturer, is not guaranteed or endorsed by the publisher.

## Footnotes

1
http://pepperhub.hzau.edu.cn/

2
https://solgenomics.net/

3
http://www.ncbi.nlm.nih.gov/

4
https://www.arabidopsis.org/

5
http://smart.embl-heidelberg.de/

6
https://en.wikipedia.org/wiki/InterPro

7
http://www.expasy.org/

8
http://gsds.gao-lab.org/

9
https://vigs.solgenomics.net/
