# Supplementary material for: Characterization of Phytochrome-Interacting Factor Genes in Pepper and Functional Analysis of CaPIF8 in Cold and Salt Stress
Source: Front Plant Sci. 2021 Oct 25;12:746517. doi: 10.3389/fpls.2021.746517 (PMC8572859; doi:10.3389/fpls.2021.746517)
Supplement: Supplementary file 3 [file Table_3.DOC]

**Table S3.** Sequences and lengths of motifs among PIF proteins of different plant species.

| **Motif** | **Conserved amino acid sequences** | **Width** |
| --- | --- | --- |
| 1 | HNLSERRRRDRINEKMRALQELIPNCNKTDKASMLDEAIEYLKSLQLQVQ | 50 |
| 2 | DDELVELLWENGQVVMQGQGP | 21 |
| 3 | ARRSTSTKRSRAAEV | 15 |
| 4 | QEDETVPWLHYPJDD | 15 |
| 5 | MMWMGSGMAPP | 11 |
| 6 | MFPGVHQYMPPMGMGMGMG | 19 |
| 7 | KKRKTRDAEDSESQSEDAEDESADTR | 26 |
| 8 | AGDTLESIVHQATYH | 15 |
| 9 | SVGTIGSSHCGSNQV | 15 |
| 10 | QPMNMEAYNRMAALYQQQQ | 19 |
